# Challenges and opportunities in implementing and enforcing India’s loose cigarette sales ban: A qualitative stakeholder analysis

**DOI:** 10.1371/journal.pone.0316342

**Published:** 2024-12-26

**Authors:** Mayank Sakhuja, Daniela B. Friedman, Mark M. Macauda, James R. Hebert, Mangesh S. Pednekar, Prakash C. Gupta, James F. Thrasher

**Affiliations:** 1 UNC Lineberger Comprehensive Cancer Center, University of North Carolina at Chapel Hill, Chapel Hill, NC, United States of America; 2 Department of Health Promotion, Education and Behavior, Arnold School of Public Health, University of South Carolina, Columbia, SC, United States of America; 3 Department of Epidemiology and Biostatistics, Arnold School of Public Health, University of South Carolina, Columbia, SC, United States of America; 4 Healis Sekhsaria Institute for Public Health, Navi Mumbai, Maharashtra, India; West Bengal State University, INDIA

## Abstract

**Introduction:**

Several Indian states have banned the sale of loose cigarettes, and India is considering a national ban. This study examines the perceptions of policymakers, implementers, and law enforcement officials regarding the implementation and enforcement of this ban.

**Methods:**

Between May–October 2022, we conducted in-depth interviews with 26 key stakeholders involved in tobacco control in two Indian cities, Delhi (where the ban was not implemented) and Mumbai (where the ban was in effect). Participants included representatives from various government departments such as police, municipal corporations, FDA, health, and civil society organizations / NGOs such as Vital Strategies, World Health Organization, International Union against Tuberculosis and Lung Disease, and local NGOs. Interviews explored policy awareness, implementation and enforcement status, and factors influencing implementation and enforcement of the ban. Interview transcripts were organized in NVivo, and reflexive thematic analysis was conducted.

**Results:**

In Mumbai, awareness of the ban was poor among implementers and law enforcers, including FDA, municipal, and police officials, while it was higher among health department officials and NGOs directly involved in tobacco control. Participants from Mumbai expressed that the implementation and enforcement of the ban was poor and loose cigarettes were still widely available. Main barriers to policy implementation included unclear implementation guidelines, poor awareness among tobacco vendors, limited resources, and lack of stakeholder commitment for tobacco control. Participants from both the cities emphasized the need for a vendor licensing system, imposing hefty penalties, imparting health education, and a multi-sectoral approach for effective policy implementation and enforcement.

**Conclusion:**

Limited awareness of the ban and continued sale of loose cigarettes highlight gaps in policy implementation and enforcement. Effective policy implementation and enforcement requires raising awareness regarding the policy, adopting a tobacco vendor licensing system, and establishing clear implementation guidelines involving a multi-sectoral approach.

## Introduction

About 11% of the Indian population aged 15 years and above are exclusive users of smoked tobacco products, with 4% being current cigarette users [1]. Loose cigarettes, where vendors sell individual sticks from commercial packs, are highly prevalent in India, with approximately 67% of cigarette users purchasing loose cigarettes at their last purchase [1]. The sale of loose cigarettes violates Section 7 of the Cigarettes and Other Tobacco Products Act (COTPA), a national law that mandates cigarette packs to depict pictorial health warnings on 85% of the total packaging area [2].

Loose cigarette sales present significant challenges to the effectiveness of COTPA provisions such as pictorial health warnings on cigarette packs, and increased taxes on tobacco products. Individuals who purchase loose cigarette sticks are less likely to see the health warnings required on cigarette packs, reducing the impact of such warnings [3–6]. Furthermore, the availability of loose cigarettes, despite their higher per-unit price, makes smoking more affordable by allowing individuals to buy in smaller quantities. This affordability undermines the intended effect of tax increases on tobacco products [7,8], as loose cigarette prices often do not rise proportionally with pack prices. Instead, they are sold at a variable markup influenced by local market conditions, which means that small tax increases on packs may not be passed on to loose cigarettes at all [9]. As a result, individuals can circumvent the full impact of tax hikes, weakening the overall effectiveness of tobacco taxation in reducing consumption.

In 2015, the Government of India proposed amendments to Section 7 of COTPA stating that cigarettes and other tobacco products must be sold and purchased in their sealed, intact, and original packaging [10]. Following this, several states, including Maharashtra, Punjab, Himachal Pradesh, Karnataka, and Chhattisgarh have implemented bans on loose cigarette sales [3,11–14]. However, many of the remaining states that represent densely populated regions with high smoking prevalence are yet to implement it. Despite strong recommendations by the World Health Organization (WHO) Framework Convention on Tobacco Control (FCTC) for banning the sale of loose cigarettes [15], existing studies that have measured compliance with India’s tobacco control laws have showed low adherence to COTPA, documenting the inadequacies in implementation and enforcement [16–20].

Policy implementation and enforcement involve multiple stakeholders who can directly or indirectly influence decision making and processes related to the regulation’s success [21]. In this study, we define policy implementation as the activities focused on the adoption and formalization of the loose cigarette sales ban, while we define policy enforcement as monitoring, ensuring compliance, and imposing penalties to uphold the ban, and consider it as a part of the overall implementation strategy. Understanding stakeholder perspectives on both implementation and enforcement is crucial for assessing their roles, interests, influence, and resources and skills they bring to affect policy outcomes [22]. In policy implementation research, stakeholder analysis can be used to understand stakeholders’ roles and potential contributions in policy processes or inform future directions for policy implementation [22]. Given the potential public health benefits of banning loose cigarettes, both in promoting cessation and preventing initiation [23], and as India moves towards a national ban on loose cigarette sales [24], analyzing stakeholder involvement can help strengthen both the implementation and enforcement of COTPA’s provisions on this issue.

Using a qualitative approach, this study aimed to understand the perceptions of key stakeholders in two cities, Delhi and Mumbai, regarding the implementation and enforcement of loose cigarette sales ban. This study is novel as the stakeholder analysis will not only aim to evaluate the existing policy ban in Mumbai (a city in Maharashtra, India) but also help advance and apply the project’s findings to cities where the ban is yet to be implemented.

## Methods

### Study design

Between May-October 2022, we conducted in-depth interviews with 26 key stakeholders, including policymakers, implementers, and law enforcement officials from two Indian cities: Mumbai, where a ban on the sale of loose cigarettes was implemented in 2020, and Delhi, where the ban had not yet been implemented at the time of data collection. These cities were selected for their diversity in geographic location and tobacco use prevalence. Mumbai has the lowest smoking prevalence among Indian states at 3.8%, while Delhi’s smoking prevalence (11.3%) is higher than the national average of 10.7% [25]. Additionally, Delhi, as the national capital, hosts key national health officials and foundations, while Mumbai, the financial capital, offers a unique perspective on implementation challenges. The inclusion of these two cities allowed us to compare findings, identify differences in implementation barriers and facilitators, and draw lessons for broader policy expansion.

### Recruitment and interview protocol

We began by compiling a list of key stakeholders authorized under COTPA to enforce tobacco control provisions in India. The initial list included personnel from various government departments (e.g., Health, Police, Food and Drugs), heads of academic institutions, and personnel from municipal corporation bodies (department that provides civic services at the city/state level). In consultation with in-country partners, we expanded the list to include key personnel from non-governmental organizations (NGOs), foundations, research institutions, and international organizations directly involved in tobacco control.

MS conducted in-person interviews with policy implementers and enforcement officials, including government officials, heads of academic institutions, and NGO officials, while also reaching out to additional stakeholders via email and LinkedIn to schedule virtual interviews. MS provided potential participants with a project introduction and emphasized the importance of their perspectives in effective policy implementation. Some agreed to be interviewed on the spot, while others requested follow-up meetings. We also contacted national-level stakeholders from the Ministry of Health and Family Welfare (MoHFW), but despite follow up emails, received no response.

Interviews were conducted either in-person at participants’ offices or virtually (over zoom or telephone call), depending on their preference. One senior municipal corporation official from Delhi opted to respond to interview questions in writing. All interviews were audio recorded with participant consent, and notes were taken during and after each interview. Participants were offered a $10 cash incentive for their participation.

### Conceptual framework

The interview guide for this stakeholder analysis was informed by the policy implementation research framework developed by Balane and colleagues (2020) [21], which examines key stakeholders’ characteristics and how those characteristics interact with one another. Interview questions were organized around four key themes: knowledge, interest, power, and position of stakeholders with respect to the ban on loose cigarette sales. Knowledge referred to stakeholders’ awareness and understanding of the policy; interest captured their motivations and perceptions of policy impact; power referred to their ability to influence policy implementation; and position reflected their support, opposition, or neutrality toward the policy.

### Data collection tool

The interview guide comprised 20 questions to explore stakeholders’ knowledge, awareness, and understanding of the loose cigarette sales ban, their roles in tobacco control, the policy’ perceived impact, and how they contributed or could contribute to its implementation and enforcement. Questions also addressed the barriers and facilitators to effective policy implementation and enforcement.

### Data analysis

All interviews were transcribed by a professional transcription service. The transcripts were organized using NVivo® [26] and analyzed using thematic analysis. A preliminary set of *a priori* codes was developed based on the interview guide to inform the analysis. Three authors (MS, DBF, MMM) independently coded one transcript, followed by a collaborative discussion to refine the preliminary codebook and add new codes as needed. MS then performed a line-by-line analysis for all transcripts and added emerging codes to the codebook [27]. Once the codebook was finalized, axial coding was used in which thematic relationships among existing codes were identified [28].

### Ethics statement

The study was approved by the institutional review board of the University of South Carolina (Pro00120549) and the institutional ethics committee of Healis Sekhsaria Institute for Public Health (FWA00019699).

## Results

In this paper, we present findings related to participants’ awareness of the policy, the status of its implementation, and barriers and solutions for effective implementation and enforcement. We also discuss participants’ current and potential contributions to the implementation process.

### Key participants

A total of 26 interviews were conducted. Participants belonged to various sectors including government departments like the Department of Health (n = 2), municipal corporations (n = 7), police (n = 4), heads of educational institutions (n = 3), the Food and Drug Administration (n = 1), and various inter/non-governmental organizations who were directly involved in tobacco control (n = 7) including the World Health Organization, Vital Strategies, and International Union against Tuberculosis and Lung Disease (Table 1). Participants were asked about their association with tobacco control and the specific role that they played in tobacco control policymaking, policy implementation and enforcement, or both.

**Table 1:** Participant characteristics (n = 26).

Department / Organization type	City	Type of organization	Role	Total (n = 26)
Health Department • Official 1 • Official 2	Mumbai Mumbai	State Government	Policymaking / Implementation	2
Hospital Research Centre • Official 1	Mumbai	Hospital Research Centre	Technical support	1
State Foundation 1 • Official 1	Mumbai	Civil society organizations / non-governmental organizations	Technical support	1
State Foundation 2 • Official 1	Mumbai	Civil society organizations / non-governmental organizations	Technical support	1
Police Department • Official 1 • Official 2 • Official 3 • Official 4	Mumbai Mumbai Delhi Delhi	Government / law enforcement	Enforcement / Implementation	4
Head of Educational Institutions • School Principal 1 • School Principal 2 • University Director	Delhi Mumbai Mumbai	Educational Institutions	Implementation	3
Private Sector • Official 1	Mumbai	Private Sector Company	Implementation	1
Food and Drug Administration • Official 1	Mumbai	State Government	Enforcement	1
National Foundation 1 • Official 1	Delhi	Civil society organizations / non-governmental organizations	Action research scientist	1
International NGO 1 • Official 1	Delhi	Non-governmental organizations	Enabler	1
Municipal Corporation Department • Municipal Health Officer • District Health Officer 1 • District Health Officer 2 • Medical Officer 1 • Public Health Inspector 1 • Public Health Inspector 2 • Medical Officer 2	Delhi Delhi Delhi Delhi Delhi Delhi Mumbai	State Government	Enforcement / Implementation	7
National Foundation 2 • Official 1	Delhi	Civil society organizations / non-governmental organizations	Facilitator	1
International NGO 2 • Official 1	Delhi	Non-governmental organizations	Technical support	1
International NGO 3 • Official 1	Delhi	Non-governmental organizations	Technical support	1

Policy implementers and enforcers included officials from the health department, police department, municipal corporations, and heads of educational institutions. We found that their primary role included undertaking field inspections, penalizing violations like public smoking and selling prohibited items, and regulating and issuing licenses to tobacco vendors. Meanwhile, participants involved as a technical support partner/enabler/facilitator/action research scientist facilitated policymaking and policy implementation. They were primarily associated with inter/non-governmental organizations / civil society organizations and were involved in generating and providing evidence for strengthening the policies, garnering support for stronger policies, mobilizing multi-sectoral support for robust enforcement, providing strategic guidance on health communications, and capacity building of implementers and enforcers.

### Participant’s awareness, and policy implementation status

Participants were asked if they were aware of the ban on loose cigarette sales and whether any such ban was implemented in their states or anywhere else in India. In Mumbai, officials from the health department, state foundations directly working in tobacco control, and a school principal were aware of the ban.

*“They issued a small circular on the ban on loose cigarettes and cited and referred to the COTPA section 7 violation and that they [loose cigarettes] do not have a health warning.”–*State Foundation 1 official from Mumbai

*“There is a rule here which says that you cannot sell loose cigarettes*. *Maharashtra government has banned selling them*, *but still it is sold here*.*”—*School principal from Mumbai

However, awareness among the implementers and enforcement officials was found to be limited in Mumbai. Officials from the police, municipal corporation, food and drug administration, and a university director reported that they had never heard of such a policy.

Those in Mumbai who were aware of the policy were asked about how the policy was operationalized and implemented in the city. Only officials from the state health department described operational strategies, including creating public awareness to sensitize the vendors, and enforcement squads that worked across departments to penalize violators.

*“Regarding this, they have awareness. The functionaries of the National Tobacco Control Program at the district level, like the consultants, and the counsellors, so, whenever they go on the field for outreach, they give health education to the people selling on the handcarts etc. Public awareness is a separate thing. So, there is also sensitization of the people who sell. They are also told the penalties that are applicable by the law. They are told that there is a fine applicable if they sell it in loose. This is the awareness that we create.”*–Official 2 from health department

*“Yeah*, *we have enforcement squads in 34 districts even at the taluka level we have those squads*, *but the enforcement squad is the combination of three departments*, *the health department*, *the police department*, *and the FDA*. *So*, *there exists the combination and contribution of these three departments*. *There exists only a little problem but otherwise*, *enforcement is very good*, *and the involvement of the police department is very nice as well*. *We have collected more than 5 crores as fines this year itself*.*”*–Official 1 from health department

Yet, despite these efforts, perceptions about implementation effectiveness varied. Officials from international NGO, state foundations, and heads of academic institutions noted that the policy was not being implemented and loose cigarettes still remained readily available in the city.

*“I don’t think it’s being implemented; you just go out and buy, try to buy. You will be able to. I mean, if you ask for two Cigarettes you will get, so, I mean that can be the best example rather than just talking things.”–*Official from International NGO 3

In Delhi, only those officials working in inter/non-governmental organizations were aware of the policy and of its implementation in Mumbai. They noted that while enforcement efforts sometimes lead to minor penalties, loose cigarettes continue to be sold and reflected on the broader issue of sustained enforcement.

*“I think some of the states did ban it. I think Maharashtra was the first state that banned the sale of loose cigarettes.”–Official from International NGO 2*

*“Well*, *the states have started to come up with these orders in addition to what the legal metrology and the central excise say but*, *the implementation is very mixed*, *and it goes in waves and it depends on*, *you know*, *just before a declaration of a smoke free or a tobacco free cities to be done*, *the ban on loose cigarette sale or its implementation comes around that time*. *So*, *there’s some people who get picked up*, *if you had open packets of cigarettes*, *so they get caught*, *but again the signs are too minimal*, *and people are just let away with very small punitive measures on upon them*, *so there’s really not much that happens*.*”–Official from International NGO 1*

### Barriers to policy implementation and enforcement

Participants described multiple challenges hindering the successful implementation and enforcement of the loose cigarette sales ban. Six main barriers emerged from the interviews conducted in both the cities, and they are presented in order from most discussed.

1. Unclear implementation guidelines

The most mentioned barrier was the lack of clear guidelines regarding the implementation and enforcement of the ban. Officials from the state foundations, hospital research system, private sector, and municipal department in Mumbai pointed out that there was limited clarity regarding how the policy was supposed to be implemented and by whom, and whether training would be provided. Officials mentioned that even though the policy was announced, no standard operating procedures were developed or shared, and there was no clarity around what the ban entailed, how fines were supposed to be levied, or what were the legal processes in case of any violation. Additionally, no trainings for such matters were provided to the implementers and enforcement officials.

*“But the order on the ban on sale of loose cigarettes that was released in September 2020 is not clear. I mean how will it be implemented? How will it be monitored? Who will report to whom? Will the police take action? What will be the fine for violating it? Nothing is clear. For example, for the ban on gutka pan masala, it is clearly mentioned in the FSSAI amendment that there will be a fine of INR 10,000, and certain period in jail….. everything is clearly mentioned… but for loose cigarettes… the simple circular that was released showed that the policymaker was not provided with any training related to the policy, there is no awareness in the community. We have been working in this field for so many years now and that circular was incomplete. If you are releasing any circular, it is important that those officers should receive the necessary training, which was not the case, and you can easily find loose cigarettes everywhere. Even though you released the circular regarding banning the sale of loose cigarettes, the implementation is very poor.”*–State Foundation 1 official from Mumbai

More specifically, there was lack of clarity regarding whose responsibility it was to implement and enforce the ban. On being asked whose responsibility it was to implement the ban, policy implementers and enforcers from both the cities mentioned that it was the responsibility of other departments, but not theirs, to implement the ban. For example, municipal corporation officials from Delhi felt the police, health, and education department should handle implementation, while the head of an academic institution in Mumbai perceived it to be the responsibility of municipal corporation officials and was not sure if they could enforce the ban beyond their campus.

*“But under your nose, I mean, selling undercover or selling by dubious means, all that probably may happen around the campus, which may create problems of enforcing the ban, but there is little that we can do outside the institute. But certainly, we will, you know, make sure that this is enforced within the campus, but we don’t know, I mean, the ban, the sale of loose cigarettes will not happen within the institute, but it can happen outside. So, I’m not really sure whether we can play any active role in that.”–University Director in Mumbai*

*“Now for enforcing this*, *tell me who can take action on this*? *Don’t bring the police into this*. *This act is not the job of police*. *It is the job of food and drugs*. *That act is majorly related to them*. *It is mainly their job*.*”*–Police official from Mumbai

Because of unclear guidelines, state foundation officials from Mumbai mentioned that policy implementers and enforcement officials from some departments placed responsibility on the other department, resulting in poor implementation and enforcement. Additionally, officials from national foundations in Delhi pointed that unclarity in implementing roles would be a major issue if a ban comes in place in Delhi.

*“For loose cigarettes, there are no clear guidelines regarding who should implement and enforce this law. So, every department pushes it on each other that this is not our work, who will do this? We already have lots of work to do, so this is the situation. So, someone should be nominated for this responsibility, they should designate this responsibility to someone, that you have to implement this policy. And if they want to take action, where should they take action? This should be also there. So, there is no proper management, and that’s why there is no implementation of the law.”*–State Foundation 2 official from Mumbai.

2. Lack of commitment for tobacco control

A recurring theme in both cities was a perceived lack of commitment to tobacco control. Policy implementers and enforcers from Mumbai, including officials from the police, FDA, municipal corporation, and head of an academic institution expressed that tobacco control was not a priority in the city as much as it should be. Police officials stated that tobacco control was not a priority for their department given their numerous other responsibilities. Similarly, a municipal corporation official mentioned that their focus was more on treating non-communicable diseases than participating in tobacco control related initiatives. Moreover, officials from the state foundations in Mumbai also pointed out that tobacco control was not prioritized and issues related to implementing the loose cigarette sales ban were being neglected by senior government officials.

*“I am telling you that it [priority] is not there. If they wanted it, then it could have been done 100% throughout India but it is not the case. It is not going to help if it is done only in Maharashtra. Just asking the state authorities to implement it does not help. We are doing it because we have to do it. We cannot say no. But if you want to do it then do it throughout India otherwise don’t do it. They can do it, but they are not focusing on it.”*–FDA official from Mumbai

*“It is not really a priority as per our department since we have to work on everything*. *We work on whatever comes our way*. *But this is necessary*. *This should be focused upon*.*”*–Police official from Mumbai

Participants from Delhi echoed similar opinions regarding low commitment for tobacco control. Policy implementers including municipal officials stated that their department did not make tobacco control a priority and that they were not directly involved in tobacco control. Officials from international NGOs felt that tobacco control was not prioritized at the national level and considered state and district level officials to be more responsive to the issue of tobacco control. However, officials from national foundations expressed lack of efforts for tobacco control by the state government in Delhi.

*“I think we have more faith in state and district, we don’t really have much hope on national support. It’s too complex and too politically compromised, so it’s always best to go to States where you find some local champions who want to make a difference.”*–Official from International NGO 1

*“At the level of Delhi Govt*. *while you know there is general support for public health measures*, *I mean I do not see too much happening at the state level in terms of tobacco control*.*”*–National Foundation 2 official from Delhi

Enforcement and NGO officials in Mumbai also stated that there was interference in policy implementation from local politicians which made implementation a challenge. It was also difficult for strong policies to pass due to policymakers’ interests in the tobacco lobby. An FDA official also described how local politicians interfered during their field visits regarding prohibited items and coerced them to not take necessary action against the violators.

*“After we go there on the field, there is local interference from the local politicians, who say that he is our person, they pressurize us so that is a problem. We anyway do our work, but it is a distraction.”*–Official from FDA in Mumbai

*“If you will see*, *policy makers*, *especially politicians do give instructions to ban this*, *or ban gutkha pan masala*, *or ban loose cigarettes*, *so they do have that intention*, *but sometimes what happens is like the social justice department and politicians of other departments have interests in the tobacco lobby*, *and sometimes they are also tobacco users*, *so strong policies can’t be passed*.*”–*State Foundation 1 official from Mumbai

3. Resource limitations

Participants also cited a lack of resources, specifically financial support, infrastructure, and manpower, as a significant obstacle to policy implementation and enforcement. Enforcement officials from the FDA and police in Mumbai described how the shortage of human resources limited their ability to carry out raids and inspections, particularly in areas with a high density of cigarette vendors. FDA official also mentioned that there were limited financial resources, and infrastructure, such as vehicles, to carry out the field visits by enforcement officials. Because of lack of infrastructure, they also referred to undertaking field visits as a risky task. As a result of lack of manpower, they described that they would collaborate with other departments, such as police, to carry out enforcement related activities.

*“If you want to raid many places in Mumbai, then you will need that much manpower. To go there… There should be good management so that you can raid many places in one go. That won’t be possible, right? If I go alone, then I will go to one stall and conduct a raid and then go to the other. This is Bandra. Here, there are so many cigarette stalls. So, these problems are bound to come.”*–FDA official from Mumbai

*“That is always there [need for more personnel]*. *Even now it is less*. *Do you know what is the strength of one police station*? *I think 230–240 is the strength that is sanctioned*, *but only 156 people are working*. *Human resource is needed*.*”*–Police official from Mumbai

The FDA official described that they did not have any financial support for undertaking field visits to seize prohibited items. They had to incur expenses themselves which were not reimbursed to them. They also brought up that undertaking such visits without a vehicle and security was risky.

*“See, we don’t get any funds for all these things. The expenses incurred on the vehicle for going and coming back. The expense incurred for bringing the stock here, and also for loading and unloading, we have to give it ourselves. We cannot claim it from the office. There is no mention of funds. It is just like that. It works this way.”*–FDA official from Mumbai

*“We form a team and go there [for inspection]*. *We don’t get a vehicle to go there*. *We don’t get any security*. *So*, *we first go to the police station*. *We give them a letter and then get the security from them*. *One or two policemen come with us in the rickshaw*. *Then we go there*. *After reaching there*, *they [violators] will try to argue that they have not done it*. *Sometimes they run away from there*. *All these things happen*. *After that registering an FIR is also a problem*. *I am telling you about the prohibited articles and not about cigarettes*. *There are difficulties*. *After that*, *if the stock is more*, *then carrying the stock from that place where we have seized it to bring it here involves so many difficulties*. *We have to look for a vehicle*. *There is no infrastructure*, *so these difficulties are there*. *If I go at 9 in the morning*, *then I reach home by 2 or 3 in the night for one raid*. *There are many challenges*.*”*–FDA official from Mumbai

*“I am telling you about my experience*. *I am telling about prohibited articles*. *I am a lady officer*. *To go there is a big thing for me*, *to go to the stall because they are criminal-minded*. *If they are selling banned products*, *then they have some connection*. *So*, *to go there is risky*.*”–*Official from FDA in Mumbai

Officials from the health department and state foundation in Mumbai also pointed to the difficulties in managing the tobacco control program related activities due to scarce manpower. They stated that for implementing the policy, there were fewer resources at the district level and below. As a result of that, they focused on multi-sectoral coordination with other government departments such as the police, for implementing tobacco control related provisions.

*“No, this policy is implemented because of the 2003 law. We are following the guidelines written in it. It talks about following a multi-sectoral approach and all. We involve other people in this because there are only 3 members in Tobacco Control Program, maximum posts in the tobacco control program are vacant. My district would not get monitored by 3 persons that’s why I have included all the departments and we get information from them.”*–Official 1 from Health Department

Municipal corporation officials from Delhi also discussed issues around paucity of health staff and that no funds were allocated to them for implementing such policies.

4. Weak monitoring and evaluation systems

Officials from national foundations in Delhi and hospital research centre in Mumbai emphasized the lack of monitoring and evaluation mechanisms for loose cigarette sales. They referred to the high density of vendors selling tobacco products in the country which made monitoring a very challenging task. They mentioned that real time evaluations or on-the-field assessments of tobacco control policies were rarely conducted.

*“And the other thing also is, an initial challenge is about checking this loose sale. It is very very difficult to control because you know in a country like ours tobacco products are something which are available at every local corner and so checking something like loose sale will be definitely a challenge.”*–National Foundation 2 official from Delhi

*“Another important thing is also about evaluation and that is again something which is not spoken as much in India as probably in some of the other countries is that when laws and legislations are enforced after a certain point in time*, *when reasonable period has been passed where you know that law has been in place*, *we don’t evaluate the level of enforcement or the impact that it has brought about*. *So*, *I think for tobacco control policies in general and even for this new policy of ban on loose sales*, *evaluating the implementation of policy is very important*.*”*–National Foundation 2 official from Delhi

Health Department officials and those from international NGOs stated that not having a proper licensing system for tobacco vendors was a challenge in the implementation of the ban. It made controlling and monitoring loose sales difficult as vendors without a license were able to freely sell tobacco products from any place in the city.

*“They are freely sold in loose. They start selling anywhere. Maharashtra is struggling with the vendor license issue. Vendor licensing, we are trying to promote from our end at the state level. If willpower is raised amongst the policymakers and bureaucrats… See, they sell it anywhere on the road on a handcart. They will take the handcart from one place to another. If the police come and takes action against them, then they shift their handcart from one place to another. We think that if vendor licensing is properly done, then they will get taxed. So, there will be control over the sale and the monitoring and supervision can be properly done.”–*Official 2 from health department

An official from Mumbai also stated that there was no working platform where complaints could be raised if someone was found selling loose cigarettes, and a toll-free number previously available for reporting violations was no longer operational.

*“Plus one more thing. The toll-free number which was given by the central government to raise complaints, that is not even operational. That’s the whole thing. Where will the general public raise their complaints then? If there is a portal and I need to reach out to someone, it is just not possible. There should be a platform available where people can raise complaints. That is the most important. There is no such platform available. It was there earlier administratively, but it even got shutdown administratively.”*–Official from Hospital Research Centre

Enforcement officials from Delhi including municipal corporation and police, head of an academic institution and state foundation official from Mumbai described that resistance from tobacco vendors and corruption among enforcement officials could further undermine monitoring and evaluation efforts. They shared that if loose cigarettes were banned, tobacco vendors would resort to selling loose cigarettes discreetly.

*“Secondly, law implementers themselves induce bias while implementing the law. Suppose they will go somewhere to enforce the law, and say they themselves smoke, so then they will ask the vendor to give them four, five or ten packets [referring to bribe]. So this is a big gap.”–*State foundation 2 official from Mumbai

5. Tobacco industry interference

Tobacco industry interference was described as a critical barrier in the implementation and enforcement of tobacco control provisions. Participants from international NGOs and state foundations stated several tactics that the tobacco industry used to delay policy approvals and implementation. These included funding political parties, offering gifts and bribes to policy officials, and raising concerns about the livelihoods of tobacco vendors. They also described that tobacco control policies received a lot of pushbacks from the tobacco industry, and that the ban on loose cigarette sales would face similar pushbacks from the industry.

*“And the interference of tobacco industry is so much like….at the time of new year…representatives from ITC company will gift new year calendars to these officials, and then that officer will never step forward to implement that tobacco control law. There is a lot of tobacco industry interference in Maharashtra, especially with ITC.”–*State foundation 1 official from Mumbai

6. Lack of awareness about the policy

Multiple stakeholders from the health department and state foundations in Mumbai pointed to the fact that awareness regarding the policy was low among tobacco vendors and public; only those who were involved in tobacco control were aware of it since they advocated for it. They mentioned that it was not possible to assess the impact of policy as vendors were still selling loose cigarettes due to lack of awareness.

*“From the perspective of loose cigarettes, I would say, the most important thing which I feel is that, the ban which has been implemented on loose cigarettes, that ban has not been portrayed that effectively from the government’s side. As per COTPA, people cannot consume tobacco within the 100 yards radius of an educational institution, and people are aware of it now. The thought or the severeness behind it is something that people do not know. But somewhere down the line what I feel is, this idea that loose cigarettes are banned, has not reached people properly. Like, this rule that you are not allowed to sell cigarettes to minors, tobacco vendors know about this, but the fact that loose cigarettes are banned, this is something that tobacco vendors do not know. And tobacco vendors are not aware about it because there is no vigilance authority supervising tobacco vendors which can inform and update them about these policies which are drafted.”*–Hospital Research Centre official from Mumbai

*“We have launched the notification but if you go into the field then you would see that people are not aware of this*, *they still sell loose cigarettes*. *Specially paan shops*, *or any small shops sell it*. *So*, *our target is to make them aware*. *We should tell those who sell chocolate or any biscuit to not sell cigarettes because they [kids] would come to buy these products*, *and they will notice that cigarettes are being sold so they may end up trying the cigarette and get into the habit of smoking*. *That is why awareness is important*. *These activities happen but we can’t calculate the impact that has been there*. *But there has been some impact*, *not 100% but some for sure*.*”–*Health Department official 1 from Mumbai

### Facilitators for policy implementation and enforcement

We asked the participants about the potential facilitators or solutions for effectively implementing and enforcing the ban on loose cigarette sales. Several key facilitators emerged, including imposing heavy fines and penalties, issuing tobacco licenses to vendors, reducing tobacco industry interference, promoting health education about the harmful effects of smoking, and improving multi-sectoral coordination between implementing departments.

1. Stronger implementation and enforcement mechanisms

Participants strongly emphasized that strict implementation of the ban on the sale of loose cigarettes was needed. Policy implementers and enforcers from both the cities, including municipal officials, police officials, heads of academic institutions, and officials from the health department, stated that heavy fines and penalties must be imposed on tobacco vendors for the policy to have an impact. Without the presence of fines, tobacco vendors would continue to sell loose cigarettes. They noted that fines must be substantial and enforcement thorough, with a focus on deterring vendors through fear of the consequences. Participants also cited the effectiveness of strict enforcement in other areas that significantly reduced the number of violations for running a red light or smoking in public transport.

*“If it is to be done then it has to be stringent. If it is a non-cognisable offence then it will not have any impact. The person will easily go by. He will pay the fine and again open the shop and start selling again. He should have that fear that he cannot sell loose.”–*Police official from Mumbai

*“The law should be quite stringent for this*. *There should be hefty fines*. *If somebody gets caught then there should be a hefty fine*. *The way if someone jumps a red light then nowadays there is a huge fine*. *Fewer people jump*. *Similarly*, *if there is a fine of Rs*.*200/- and if there is a fine of Rs*.*2000/-*, *if you sell loose cigarettes then there should be a fine of Rs*.*2000/- on you or Rs*.*5000/- fine*. *Then the shopkeeper will think that I don’t want to get into this mess*. *Why should I sell*? *I will lose all the profit that I made on that day*. *This is also there*. *The fine and the punishment should be at a deterrent level*.*”*–District health official from Delhi

2. Implementing vendor licensing

Participants from both the cities emphasized the need for having a vendor licensing system for selling tobacco products. They stated that vendors must acquire a separate license for selling tobacco products, just like a liquor store or a gas station had a license to operate. Policymakers and implementers from the health department in Mumbai stressed that it was difficult to put a check on vendors if they were selling tobacco products without having a license. They mentioned that vendor licensing would ensure that not every shop owner would be able to sell tobacco products but only those who had a valid permit. And those with a valid permit would not be allowed to sell anything except tobacco and would need to operate from a specific geographic location only.

*“I feel that vendor licensing should be universal. That is because we can ask whether the vendor is selling loose cigarettes or not, only when he has the license. If they sell without a license like the food products then it is difficult to put checks on them. If we catch him at one place, tomorrow he will go to some other place with his handcart and start doing business. If we do the vendor licensing as we do for liquor, then it gets controlled. Similarly, if we control the sale of tobacco as well with proper licensing then there will be proper checks.”*–Health Department Official 2 from Mumbai

Officials from Delhi added that licenses should be revoked for vendors who violate the law, helping curb the informal economy where tobacco is sold on streets or handcarts.

*“I will get connected to the licensing branch that issues the license to them. If a person is found violating the act, his license should be revoked. The second thing is some people work without a license so they have to be cleared from that place. You might see many of them selling on the roadside.”*–District health official from Delhi

3. Minimizing tobacco industry interference in policymaking

State foundation officials in Mumbai noted that interference from the tobacco industry must be reduced to ensure successful implementation of the loose cigarette sales ban. They recommended creating guidelines for how policymakers and implementers interact with tobacco industry representatives, aiming to prevent undue influence.

*“I believe that interference should be reduced and for that instructions should be given at the department level, that if you want to meet the representative of the tobacco industry then there should be a proper guideline from the department, or those guidelines should be made at the CM or the chief secretary level. And those guidelines are currently not there. Because anyone will then go and meet the FDA commissioner or the director of the health department and then there is interference. So, all that will not happen and yes, behind this, there is a political agenda, and we have seen that it delays the implementation and enforcement of any new policy. To ban gutka pan masala, it took a lot of time.”*–State Foundation 1 official from Mumbai

4. Enhancing health education and awareness

Stakeholders from both the cities stressed that the enforcement alone of the loose cigarette sales ban would not be sufficient. They mentioned that the ban should be accompanied by comprehensive public health education campaigns to raise awareness about the harmful effects of smoking. Participants suggested that educational efforts be the ultimate goal to complement enforcement.

*“If you want to ban it then, realistically, social awareness is most important. You have to make people aware that what is the harm of cigarette smoking and what are the diseases that you may suffer from. They should make the law stringent. A combination of the two is required–awareness and law.”*–District health official from Delhi

5. Appointing a designated officer for tobacco control

Participants from the health department and NGOs in Mumbai stressed the need for a designated officer for overseeing tobacco control efforts, which would involve coordinating and monitoring tobacco control programs, ensuring effective implementation and enforcement of tobacco control provisions.

*“There is a need for a nodal officer, if you have to collect fines from every district then there should be a proper mechanism for that. Monitoring is usually a challenge for COTPA implementation, so there should be a proper monitoring team and monitoring officers, which is currently missing.”*–State Foundation 1 official from Mumbai

*“Yeah*, *special enforcement is needed*, *power is also required*, *and enforcement too*. *But for the health department*, *more than enforcement and power*, *we need a dedicated person*. *See*, *I look after 9 programs*, *and tobacco is one of the small programs*. *So*, *if there are separate State Program Officers for every program*, *the programs could be implemented more effectively*.*”*–Health Department official 1 from Mumbai

6. Fostering coordination and collaboration among stakeholders

Participants strongly emphasized the need for and importance of a multi-sectoral approach for implementing and enforcing the loose cigarette sales ban. Given limited resources and multiple responsibilities, health department officials, enforcement officials, and state and national foundation representatives from both the cities believed that improved collaboration between government departments would significantly enhance policy enforcement.

*“Yes, it can be done. Definitely, it [better implementation] is possible. The police have a lot of information. They have more informers. So, the raid can be done collectively taking along other departments.”*–FDA official from Mumbai

Policy implementers and enforcement officials also suggested involving other institutions, such as resident welfare associations, schools, and the general public, in tobacco control efforts. Moreover, state foundation officials from Mumbai believed it was equally important to involve and engage with civil society organizations for their expert guidance on policymaking and implementation. Additionally, some participants advocated for expanding the authority to impose fines to school teachers, municipal officials, and community leaders to relieve the burden on police.

*“If a teacher in school is good, he should be given permission that he can fine someone. Some officer from health centre should be permitted to implement fine. So many control measures can be implemented. The reason why there is a problem now is that only a police officer is putting fine. If we have 10,000 people and 10 people are controlling them, then how would things work.”*–School principal in Mumbai

*“There are many societies*. *Suppose there is a society where there are 500 residents*, *500 families are residing*. *There will be an RWA in that society who looks after the day-to-day administration*. *They look after the housekeeping*. *They look after the electrical problems*. *They should ensure that such type of activity*, *they do not get sold loose*, *around society*, *they can approach the police or they can stop them from selling*. *This type of public mobilization is required*.*”*–District health official from Delhi

Municipal corporation officials from Delhi, along with representatives from international NGOs emphasized that to ensure efficient collaboration and coordination between multiple stakeholders, capacity building was considered crucial where each stakeholder would have the opportunity to learn their specific responsibilities to avoid confusions or lags in policy implementation. Implementers from Delhi mentioned that if they received proper guidance from the state or central government and worked as a team with other departments, then they would not face any difficulties in implementing the ban.

### Contributions to the implementation and enforcement of the policy

We asked participants to describe how they and their organizations had contributed, or could potentially contribute, to the implementation and enforcement of the loose cigarette sales ban. Notably, only officials from the state health department in Mumbai reported having already contributed, primarily through capacity building efforts and enforcement actions. Other stakeholders highlighted how they could play an essential role in the future. NGOs indicated that they could support the policy by providing capacity building training, generating scientific evidence through monitoring and evaluation, and advocating for public and political support. They also emphasized their role in raising awareness and mobilizing communities to back the ban. Enforcement officials from the police suggested that they could contribute by providing security for other enforcement agencies, assisting in raids, and conducting investigations to ensure compliance with the law. Educational institutions highlighted their potential in imparting health education and undertaking advocacy campaigns to increase public awareness about the harmful effects of tobacco use. A more detailed breakdown of the contributions, both actual and potential, from various stakeholders is provided in Table 2.

**Table 2:** Potential and actual contributions of key stakeholders to the implementation and enforcement of the loose cigarette sales ban.

Department	Contribution	
	Mumbai	Delhi
Health Department	Capacity building and enforcement	-
Hospital Research Centre	Capacity building of enforcement officials with the objective of health education.	-
State Foundation 1	Advocate and follow up with the ministry for policymaking; Involve school children in advocacy activities.	-
State Foundation 2	Capacity building of enforcement officials.	-
Police Department	Public awareness–discuss the issue with schools and colleges; Conduct investigations into case of violations; Provide security to other enforcement officials	Conduct inspections
Heads of Educational Institutions	Impart health education to children and through them to the community; Undertake advocacy campaigns; Encourage postgraduate students to undertake research studies for generating more evidence for supporting the policy.	Impart health education through school children via street plays and public rallies.
Private Sector	Advocate for the policy; capacity building of communities and enforcement officials.	-
Food and Drug Administration	Conduct inspections; issue challans / fines	-
National Foundation 1	-	Improve public awareness by sensitizing the community members; Monitoring and evaluation.
International NGO 1	-	Provide technical support for vendor licensing.
Municipal Corporation Department	Medical officer in charge of a primary health centre takes no responsibility.	Undertake field visits for inspections and issue challans/fines or seal the shop if violations are caught; Seize loose cigarettes; Control vendors through regulating license issuance; IEC activities.
National Foundation 2	-	Garner support for the policy; Sensitize young people and vendors on the policy; Monitoring and evaluation.
International NGO 2	-	Design and sustain health communication campaigns; Messaging on social media around the policy targeted towards policymakers; Create support through public agenda setting.
International NGO 3	-	Generate and disseminate scientific evidence.

## Discussion

Our in-depth interviews with stakeholders in two Indian cities regarding the loose cigarette sales ban identified a number of key issues around policy implementation and enforcement in Mumbai, a city where the ban has been in place since 2020. Stakeholders identified several barriers to policy implementation, which we categorized into: (1) unclear implementation guidelines, (2) lack of commitment for tobacco control, (3) resource limitations, (4) weak monitoring and evaluation systems, (5) tobacco industry interference, and (6) lack of awareness. Finally, the main facilitators or solutions for policy implementation that stakeholders identified were (1) stronger implementation and enforcement mechanisms, (2) vendor licensing, (3) minimizing tobacco industry interference in policymaking, (4) health education and awareness, (5) designated officer for tobacco control, and (6) coordination and collaboration among stakeholders.

Our study found that even though the loose cigarette sales ban was proposed in 2020 in Mumbai, awareness among policy implementers and enforcement officials was found to be low. Participants from Mumbai mentioned that the policy was not being implemented and that loose cigarettes were still widely available in the city. Other studies conducted in India have found that awareness and understanding of COTPA provisions among implementers and law enforcement officials have generally been poor which is considered a major impeding factor in effective policy implementation [29,30]. Poor awareness levels also signifies lack of training and capacity building opportunities for implementers and enforcers which eventually results in failure or delay in initiating action for violations [29]. Literature suggests that awareness and knowledge about the policy was linked to the stakeholders’ level of interest in the policy, where policy implementers who were unaware of the policy may have lower interest in its implementation [21]. A similar stakeholder analysis focused on understanding perceptions of stakeholders regarding a universal health insurance policy in Ghana also suggested that lack of stakeholder’s understanding of the policy acted as a hindrance in successful policy implementation as low awareness levels affected stakeholders’ interest in the policy [31].

Participants from Mumbai mentioned unclear implementation guidelines as the most common barrier to the implementation of the loose cigarette sales ban. Unclear implementation guidelines led to lack of clarity about implementation roles of various stakeholders and lack of knowledge about how the policy was supposed to be operationalized. Study findings are consistent with the global literature on tobacco control policy implementation. Study conducted by Mohamed and colleagues (2018) to assess facilitators and barriers in the formulation and implementation of tobacco control policies in Kenya stated unclear roles among members of the tobacco control unit as a barrier in tobacco policy formulation and implementation [32]. Similar findings were reported by Astuti and colleagues (2020) that unclear roles and responsibilities of tobacco control stakeholders in Indonesia led to delays in effective policy implementation [33].

WHO-FCTC focuses on the importance of strong political commitment for successful tobacco control policy formulation and implementation [15], which has been described as a barrier for implementing the loose cigarette sales ban. Participants from both the cities described that tobacco control was not prioritized by their organizations and that political leaders interfered in the policy implementation process. Evidence suggests that tobacco control has always been a low priority issue not only in India but also in other developing and developed countries [30,34–36]. Strong leadership and political commitment has been described as a key facilitator for tobacco policy formulation and implementation in Kenya [32]. The significant decrease in the smoking rates in Turkey has also been attributed to Turkish government’s sustained political commitment to tobacco control [37]. In addition to lack of prioritization for tobacco control [30], lack of resources, including financial support, infrastructure and manpower, has been a long standing barrier for effective tobacco control policy implementation in India [30,38] and in other low-and-middle income countries [36].

Stakeholders described that for implementing the ban on the sale of loose cigarettes, a strong monitoring and evaluation system was lacking. Loose cigarette sales could only be monitored if there was a robust tobacco vendor licensing mechanism. Not having a vendor licensing system in place meant that anyone, be it a grocery store or a street vendor, could sell tobacco products illegally without a license resulting in high density of tobacco vendors in the neighborhood. Studies conducted in India have found that tobacco vendor density was notably high [39]. Easy access to tobacco retailers and the retail environment in a neighborhood significantly influences tobacco use, especially among youth [40]. A study found that school children in neighborhoods with high tobacco vendor density reported increased risk of consuming smokeless tobacco [41]. The government should prioritize and implement tobacco retail licensing and learn from the experiences of states, such as Himachal Pradesh, where tobacco retail licensing has already been institutionalized to complement the ban on the sale of loose cigarettes and has remarkably reduced the availability of tobacco products [42]. Vendor licensing will prevent vendors from selling other food items with tobacco products and reduce vendor density and access to tobacco products.

Even though policy implementers described resistance from tobacco vendors as another potential barrier to effective policy implementation, cross sectional studies conducted in other states of India where loose cigarettes were banned found that most vendors admitted that if the loose cigarette sales ban was properly enforced, they would stop selling loose cigarettes [43]. Finally, interference by the officials of tobacco industry in policymaking and implementation was another potential barrier reported. This finding aligns with the global policy implementation literature. Tobacco industry delayed implementation of tobacco control policies in Kenya through instituting legal suits or by bribing senior officials [32]. Similar findings were reported from Argentina, Malawi, Colombia, and other low and middle-income countries [44–47].

Echoing the solutions participants proposed for overcoming barriers to implementing the loose cigarette sales ban, Persai and colleagues (2016) stressed that strengthening tobacco control policies requires integrating health education strategies with community-based campaigns to effectively reduce tobacco use [30]. FCTC recommends participation of multiple stakeholders, including civil society organizations, for formulating and implementing tobacco control provisions [15]. Studies focused on tobacco control in India have recognized the need for establishing a national coordination mechanism for tobacco control [38]. India can learn lessons from other low-and-middle income countries such as Brazil, who had established a similar model and had become a leader in controlling and regulating tobacco products [38]. Kenya too benefitted from a central coordination mechanism that was a facilitating factor in the tobacco control policy formulation process and ensured representation from multiple stakeholder groups [32]. Such mechanisms would ensure collaboration and coordination among various stakeholders such as NGOs, civil society organizations, and government departments at the state and national levels authorized for enforcing COTPA provisions. A multisectoral approach will help in leveraging knowledge, reach, and resources, which has been described as an important barrier in implementation. Study participants suggested that to reduce tobacco industry interference in policymaking and implementation, guidelines must be drafted regarding how policymakers should meet with tobacco industry officials. Similar solutions have been suggested in other studies advocating for adopting a code of conduct by government officials to minimize complicit connections with industry officials [33].

Finally, findings of this study highlight the diverse and critical roles that stakeholders can play in the implementation and enforcement of the loose cigarette sales ban. Participants’ responses will help in the process of assigning specific and clear roles for policy implementation. Despite the willingness of many stakeholders to contribute to the policy’s implementation and enforcement, several barriers remain, including limited resources, lack of interagency communication, and unclear policy guidelines. Strengthening communication channels between stakeholders and providing formal structures for collaboration could greatly benefit enforcement efforts. Additionally, NGOs are an important part of health diplomacy globally, and have the community reach necessary for effective policy advocacy at the local, national, and international level [48]. NGO officials from our study expressed readiness to support capacity building, advocacy, and monitoring and evaluation. Ensuring that NGOs and other stakeholders are provided with the resources and mandates to actively engage in capacity building, health communication, and advocacy can transform these potential contributions into real and sustained actions. The government must bring together all these stakeholders and design detailed implementation guidelines for implementing and enforcing the ban on the sale of loose cigarettes.

Despite its strengths, this study has some limitations. Because the interviews were conducted in Mumbai and Delhi, the findings may not be generalizable to rural areas or regions in the South, Northeast or Northwest of India. Additionally, the exclusion of key national level stakeholders, particularly those in the Ministry of Health & Family Welfare, may have limited our ability to capture a more comprehensive view of policy implementation at the national level. Finally, the absence of participants from other relevant departments, such as the Excise and Home departments, may have left out important perspectives on the enforcement of the ban.

## Conclusion

This study provides valuable insights into the challenges and potential solutions for implementing and enforcing the loose cigarette sales ban in India. Through in-depth interviews with key stakeholders, we identified critical barriers, including unclear implementation guidelines, resource limitations, and tobacco industry interference. However, the study also highlights key facilitators, such as stronger enforcement mechanisms, vendor licensing, and enhanced coordination among stakeholders, which could significantly improve policy implementation and enforcement.

The strength of this study lies in its exploration of the practical, on-the-ground challenges faced by policy implementers and enforcers, as well as the diverse roles that stakeholders, such as NGOs, law enforcement, and educational institutions can play in advancing tobacco control efforts. Our findings not only underscore the need for multi-sectoral coordination but also demonstrate the importance of building capacity and raising awareness about the policy to ensure more effective policy implementation.

Future research should focus on developing tailored dissemination strategies to raise awareness and policy support among different stakeholder groups, and tobacco vendors and users. Moreover, targeted communication is also needed to address the specific challenges identified in this study. Additionally, more work is needed to assess the effectiveness of stakeholder coordination mechanisms and their respective roles in policy implementation and enforcement. Future exploration of policy dissemination strategies across different Indian regions will be critical to ensuring that the loose cigarette sales ban is effectively enforced at the national level. By addressing these gaps, policymakers and public health professionals can better align policy objectives with real-world implementation needs.
